# Intermittent Alien Hand Syndrome and Callosal Apraxia in Multiple Sclerosis: Implications for Interhemispheric Communication

**DOI:** 10.1155/2014/873541

**Published:** 2014-02-18

**Authors:** A. Lunardelli, A. Sartori, P. Mengotti, R. I. Rumiati, V. Pesavento

**Affiliations:** ^1^Rehabilitation Medicine, Ospedali Riuniti, Trieste, Italy; ^2^Department of Medical Sciences, University of Trieste, Italy; ^3^Neuroscience Area, SISSA, Trieste, Italy

## Abstract

We report a case of a 47-year-old woman with 35-year history of multiple sclerosis, who showed alien hand signs, a rare behavioural disorder that involves unilateral goal-directed movements that are contrary to the individual's intention. Alien hand syndrome has been described in multiple sclerosis (MS) only occasionally and is generally suggestive of callosal disconnection. The patient presented also with bilateral limb apraxia and left hand agraphia, raising the possibility of cortical dysfunction or disconnection, in addition to corpus callosum and white matter involvement. Her specific pattern of symptoms supports the role of the corpus callosum in interhemispheric communication for complex as well as fine motor activities and may indicate that it can serve as both an inhibitory and excitatory function depending on task demands.

## 1. Introduction

The alien hand syndrome refers to a rare behavioural disorder involving a variety of complex, goal-directed activity in one hand that is not voluntarily initiated [1, 2]. These phenomena are still not consistently nor precisely defined, and several lesion sites have been reported responsible for its appearance, including the supplementary motor area, anterior cingulate, corpus callosum, and/or posterior parietal cortex [3]; thus, the anatomic heterogeneity may explain its various clinical manifestations. There are two main forms, the callosal and frontal variants, although a posterior one has been recently described involving the posterior parietal cortex [4]. The frontal subtype generally involves the medial premotor region, including the supplementary motor area, anterior cingulate, medial prefrontal cortex, and the anterior corpus callosum and is typically characterized by reflexive grasping, impulsive groping, and compulsive tool manipulation [5]. By contrast, patients with lesions confined to the corpus callosum show mostly diagonistic dyspraxia or intermanual conflict phenomenon, a peculiar dissociative behaviour generally of the left hand, in the absence of pathological grasping phenomena, in which the hand often acts at cross-purposes to the right [5, 6]. More specifically, the term diagonistic dyspraxia refers to involuntary movements of the left hand which act in an opposite way to the actions executed by the right hand; for example, a patient puts clothes on with the right hand, but pulls them off with the left hand. Patients with diagonistic dyspraxia may also show other abnormal behaviors in the left hand during right hand tasks or during bimanual tasks, such as nonantagonistic, irrelevant movements to the right limb, and the occasional inability to move the left hand at will during a bimanual task. The syndromic specificity of diagonistic dyspraxia has been emphasized as well as its association with lesions to the posterior end of the body of the corpus callosum, especially in its ventral part, without the necessity of extracallosal damage [6].

Alien hand phenomena are mainly due to ischaemic or haemorrhagic stroke involving the anterior cerebral artery. Here we report a single case study of a 47-year-old woman, with 35-year history of multiple sclerosis (MS) who presented with alien hand signs. Alien hand has been described in MS only occasionally, generally associated with callosal involvement [7, 8]. Even though inflammatory demyelination and axon damage in the corpus callosum are prominent features of MS, clinical manifestations of alien hand or other signs of callosal disconnection, such as callosal apraxia or unilateral agraphia, have been scarcely reported. Therefore, in this report, alien hand clinical characteristics in a MS patient are described in an attempt to clarify the role of the corpus callosum in generating motor disorders as a result of the lack of hemispheric integration.

## 2. Case Report

DA, a 47-year-old right-handed woman with 13 years of education, who worked as town employee, received the diagnosis of relapsing remitting MS at the age of 32, with the postulated onset of the disease at 12 years. She has a familial history of inflammatory bowel disease, but no previous or concomitant diseases were reported. Her last relapse goes back to the age of 35 and during the last 10 years she has gradually developed progressive paraparesis and bladder dysfunction, suggesting a secondary progressive MS. Moreover, at the age of 47 she developed a focal epilepsy secondary to MS cortical lesions. The patient's expanded disability status score (EDSS) at the time of referral was 6.5; concomitant medications were baclofen and levetiracetam.

DA was referred for neuropsychological evaluation because of intermittent and bizarre writing disorders (i.e., at times her right hand would not respond or would write letters or digits different from intended ones). During the initial interview she described additional peculiar behaviors of her left hand frequently interfering with the activity of the other hand; for example, when she was opening a door or drawer with her right hand, she simultaneously pushed it shut with the left hand; on other occasions, she would open her pocket with the right hand but immediately her left hand would close it, or when choosing from the closet a pullover to dress, instead of taking the preferred one, sometimes she would find her left hand getting another, even from a different shelf. These behaviors did not always occur in the same situations and her left hand usually cooperated well with the right. She also reported occasional inability to move either the left or right hand at will during a task. Thus, her symptoms suggested the presence of alien hand signs, in particular of diagonistic dyspraxia,the core symptom of the “callosal variant.” It was also expected to detect additional signs, typical of interhemispheric disconnection, on neuropsychological testing, such as difficulty in writing with the nondominant hand, left hand apraxia, dominant hand constructional apraxia, and tactile anomia [5].

## 3. Neuropsychological and Neuroimaging Findings

A comprehensive neuropsychological assessment aimed at obtaining a general cognitive profile was also performed, and DA was systematically evaluated on unimanual and bimanual motor tests to provoke conflict between the two hands. On neuropsychological testing (see Table 1) significant slowing of information processing speed and difficulties in reasoning and poor self-monitoring abilities emerged; such cognitive defects are quite common in MS [18].

On motor testing, however, a conflict between the two hands never became evident (see Table 2); DA exhibited no grasp reflex or forced groping in either hand, her constructional ability was well preserved for both hands, and objects were correctly recognized, named, and manipulated; thus she did not show ideational apraxia either.

DA did show left hand agraphia and bilateral limb apraxia. More specifically, she showed difficulty in writing with her left hand in response to dictation, committing grapheme perseverations (e.g., c*u*oceva → c*o*oceva) and letter substitutions (e.g., conoscen*z*a → conoscen*t*a; nipoti → ni*d*oti) when writing words and sentences; no major problems emerged instead when writing nonwords, although the only mistake she made was similar in nature (e.g., sterpan*z*i → sterpan*t*i). She was aware of her errors as they were occurring, but after initiating an erroneous movement she was not able to correct or inhibit it. In addition to the reasonable clumsiness due to lack of experience in writing with the nondominant hand, there were obvious paragraphias that she could not avoid committing, hardly explicable simply with left hand disadvantage in a right-handed person. Indeed, when asked to copy words or digits, all items were copied correctly with both hands, and the left hand could draw well geometrical figures, further corroborating the hypothesis that it was not merely a question of deficiency in motor control.

**Figure 1:**
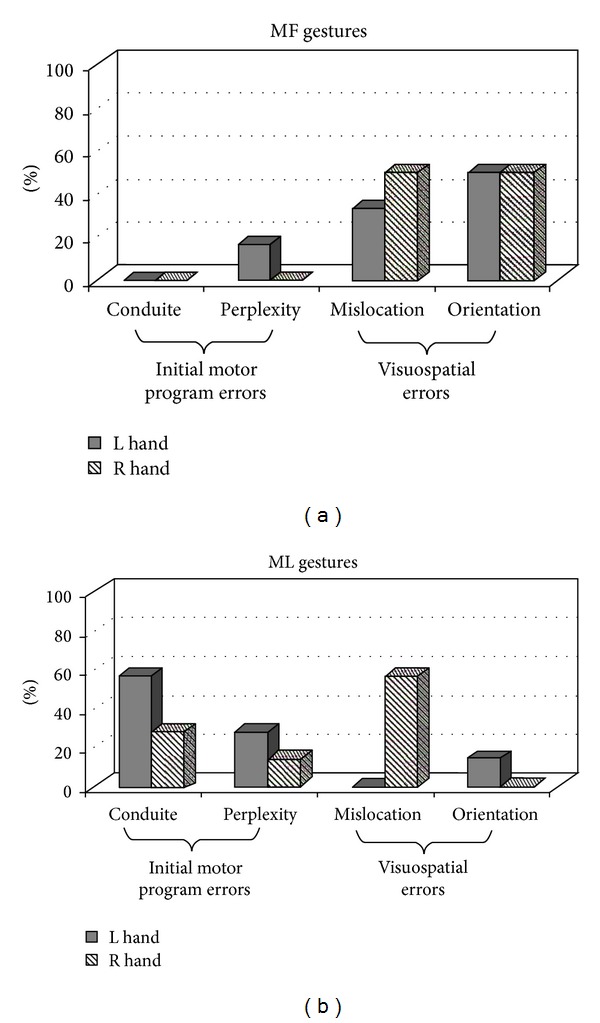
Error analysis of meaningful (MF) and meaningless (ML) gesture imitation.

As far as limb apraxia is concerned, DA was asked to reproduce 18 meaningful (MF) and 18 meaningless (ML) gestures to assess ideomotor apraxia [10], and her performance was recorded and subsequently scored by four independent raters. Interestingly, this resulted in that she was defected with both hands when copying ML postures and was impaired with her left hand when reproducing MF actions, while performing at the cutoff score when acting with the right limb (cutoff score for MF actions = 32; cutoff score for ML actions = 31 (see [10])). In order to understand which mechanisms or processes were disrupted during imitation, a qualitative analysis of DA's errors (see Figure 1) was carried out based on criteria used in previous studies [19] showing that visuospatial mistakes were prevalent with MF gestures for both hands (as indicated by the frequency of mislocations and errors on movement orientation) and their incidence was also statistically significant (*χ* ^2^: 10.00, df: 1, *P* = 0.002). In contrast, with ML actions, errors on initial motor planning were significantly more frequent (*χ* ^2^: 4.32, df: 1, *P* = 0.038) and they were mainly committed by the left hand while with the right hand mostly mislocations were made, whose occurrence resulted to be also significant compared to motor errors (*χ* ^2^: 3.94, df = 1, *P* = 0.047).

**Figure 2:**
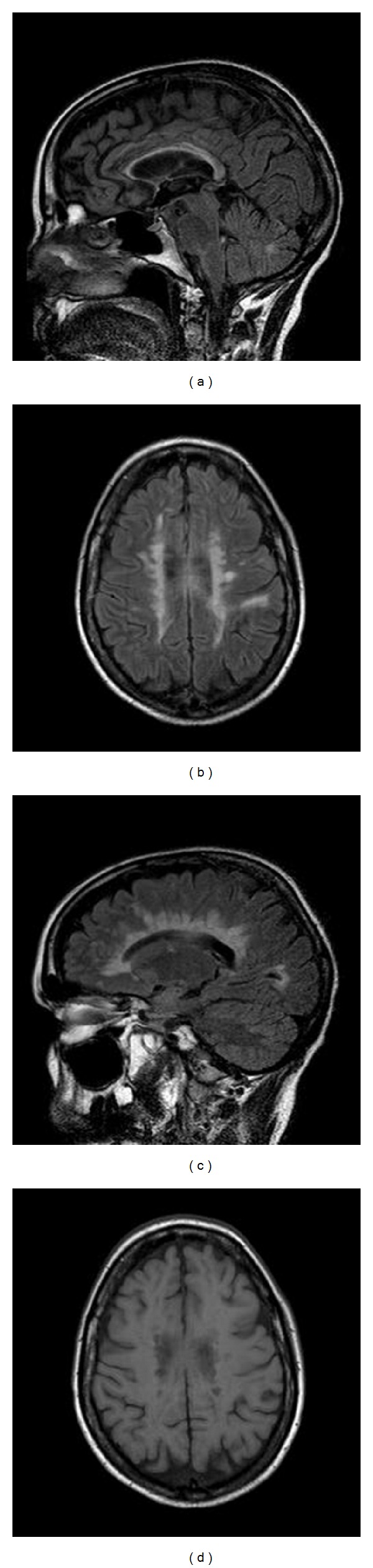
Sagittal (a), axial (b), and parasagittal (c) FLAIR MRI scan showing corpus callosum involvement. T1 axial scan (d) showing the presence of numerous T1 hypointense lesions (“black holes”).

As far as neuroimaging findings are concerned, magnetic resonance imaging (MRI) confirmed confluent lesions in the white matter and a massive involvement of the corpus callosum, particularly affecting the caudal portion, thus possibly responsible for alien hand symptoms (see Figures 2(a), 2(b), and 2(c)). Furthermore, as can be seen in T1 MRI scan (see Figure 2(d)), T1 hypointense lesions, the so-called “black holes” [20] suggestive of areas of severe tissue damage, were also present.

## 4. Discussion

Even though DA was still experiencing conflicting motor behaviour when acting spontaneously, we were not able to elicit alien movements of the hand in a controlled way. Usually the so-called automatic/voluntary dissociation refers to patients that may produce gestures correctly in ecological conditions when they act spontaneously, but not in testing sessions, when they have to execute gestures upon request [21]. DA instead, as a sort of *inversed* automatic/voluntary dissociation, could execute goal-directed actions correctly upon request and would perform oddly only in everyday life. According to classic models of motor control, motor behaviours lie on a continuum of being externally evoked (exo-evoked) or internally driven (endo-evoked). As such, her diagonistic dyspraxia may reside more on the circumstances under which actions are evoked than in the nature of the act itself, causing dissociative behaviours more likely to be driven by exo-evoked contingencies and leading the alien limb to be disproportionately compelled by environmental stimuli (exo-evoked) rather than by goals (endo-evoked) [22]. In addition, alien hand behaviours have been reported to be increased in conditions of fatigue, anxiety, or under reduced attentional control [23]; all aspects are known to characterize patients affected by MS, and are therefore likely responsible for its intermittent appearance.

Unilateral agraphia of the left hand, a classical symptom of interhemispheric disconnection was indeed observed. Left hand agraphia can be explained in terms of left hemisphere language mechanisms becoming disconnected from right hemisphere motor areas that control movement of the left hand as a consequence of damage to the corpus callosum [24]. In DA, errors appeared only when writing to dictation but not when copying, suggesting that interruption of transcallosal interhemispheric communication interfered with the transfer of left hemisphere auditory-verbal information required for guiding the left hand in performing the movements necessary to execute graphomotor patterns and controlled by right frontal areas. Unfortunately, writing skills were not systematically tested as they were only functional to confirm symptoms of disconnection; the number of items was both small and dissimilar across tasks; therefore specific hypotheses concerning why DA committed more errors when writing words and sentences than when composing nonwords cannot easily be drawn. However, because errors should emerge after the lexical system, there is no reason to expect such a disparity; they might simply reflect an effect of the frequency of words (*N* = 10) with respect to nonwords (*N* = 5).

We failed however to show classical “callosal” unilateral apraxia of the nondominant limb rather, depending on the type of action to be imitated, DA committed apraxic errors with both hands. From a quantitative point of view the possibility of a false positive result cannot be completely ruled out (as with either hand the level of performance was close to the cutoff value); nevertheless the qualitative analysis carried out on DA's imitative responses may strengthen these findings. Unlike healthy controls, who tend to make only orientation movement mistakes with whatever gestures they are asked to reproduce [10], errors committed by DA were different depending on the type of the actions to be imitated rather than on right and left hand abilities as it may be expected in a right-handed subject. This error dissociation is consistent with other neuropsychological reports [19, 25] and resembles apraxic patients' behaviour. Coexistence of extensive damage to the white matter as indicated by imaging findings may explain the presence of bilateral limb apraxia; DA's symptomatology in fact may not be attributed to interruption of callosal fibers per se, but also to concomitant plausible disconnections between corticocortical and corticosubcortical areas caused by white matter disruption.

Dual-route models of action processing [26, 27] assume that different mechanisms would be engaged depending on the content of the action to be imitated, with the ventral stream of the left hemisphere mostly involved in case of familiar, meaningful gestures through activation of their stored representations, and the contribution of the dorsal pathway of the right hemisphere in case of novel, meaningless acts, which allows the conversion of any visually presented action into a motor output. Within this framework, DA's impaired imitation may arise from a disconnection between the dorsal and ventral visual pathways; stored representations from the ventral stream of MF actions may not access the sensorimotor control processes of the dorsal stream leading to visuospatial errors; similarly, when copying ML postures with the left hand, the required perceptuomotor transformations of the dorsal stream may not receive input from premotor/motor areas, causing motor program errors. In contrast, when acting with the right hand, mostly mislocation errors emerged. This result may be consistent with a recent hypothesis [28] according to which imitation of ML hand positions would be controlled by the left hemisphere, whereas orientation of the hand may require participation of the right hemisphere. Although most studies show a left-brain hemisphere dominance for praxis, DA's patterns of errors argue for a relevant contribution of the right hemisphere and support a model of praxis distributed across hemispheres instead of solely left sided as classically postulated [29].

## 5. Conclusions 

Symptoms of callosal disconnection in MS patients have been rarely reported, although demyelinating lesions and neural connectivity abnormalities within white matter are characteristic of the disease and these findings are particularly prominent in the corpus callosum [30]. Moreover, functional impairment of interhemispheric transfer has been correlated in MS to the degree of callosal atrophy and to the severity and diffusion of white matter changes identified by MRI [31]. As a consequence, it is tempting to speculate in this case that extensive axonal loss (as shown by T1-hypointense lesions) in addition to corpus callosum and white matter involvement may be relevant to the appearance of alien hand signs. However, significant cortical pathology in addition to white matter pathology may be also present here given the co-morbid epilepsy, thus making it impossible to determine which factor or specific pathobiological substrate (e.g., axonal loss, demyelination, etc.) are more relevant.

Both alien hand signs and limb apraxia in DA suggest that the impediment of information transfer to the opposing hemisphere may result in motor disorders as a result of the lack of hemispheric integration. Disruption of coordinated hand movements that involve proximal and distal components for reaching and grabbing, respectively (thus requiring interaction between ipsilateral and contralateral hemispheres), or action impairments that require interhemispheric cooperation (i.e., combining visual-motor information of the right hemisphere with action representations present in the left hemisphere in case of gesture imitation) provide support to the role of the corpus callosum in interhemispheric communication for complex, fine motor activities as well as high-order cognitive information. However, how it mediates this information transfer remains unclear; while participation of bilateral brain regions can be seen as an excitatory function by allowing integration between the hemispheres [32], the release of unwanted movements from conscious control in case of damage to the posterior portion may indicate an inhibitory influence of an intact corpus callosum and the existence of alternative, subcortical pathways that can transfer interhemispherically even high-order cognitive information. Indeed, available research sustains both functions within the corpus callosum and there is the possibility that it may be inhibitory at times and excitatory at other times. This may be dependent on recruitment of different callosal areas and on a subcorticocortical network that balances hemispheric activation according to the task demands [32].

## Figures and Tables

**Table 1:** DA performance on neuropsychological testing (the star indicates scores below cutoffs).

Cognitive functions	NPSYCH tests	Raw score (corrected)	Range	Cutoff scores
Language	Naming	30	0–30	
	Fluency by phoneme	33	—	≤17.35
	Fluency by category	42	—	≤24

Short-term and working memory	Visual span			
	-Corsi test	4 (3.50)*	0–10	≤3.75
	Digit span			
	(i) Forward	6 (5.75)	0–10	≤3.75
	(ii) Backward	4	0–10	

Long-term memory				
(i) Verbal LTM	List of words			
	(i) Immediate recall	49 (46.3)	0–80	≤25.14
	(ii) Delayed recall	12 (11.2)	0–16	≤3.44
(ii) Visual LTM	Story recall	9.7 (8.95)	0–16	≤4.50
	Delayed recall of Rey's figure	13 (13.25)	0–36	≤9.46

Attention and information processing	Trail making test (TMT)			
	(i) Part A	93′′ (83)	—	≥94
	(ii) Part B	148′′ (111)	—	≥282
	Symbol digit modalities test			
	-Oral form	21*	0–100	

Executive functions	Raven's CPM	33 (30.8)	0–36	≤18.96
	London tower	28	0–36	
	Modified card sorting test			
	(i) Categories	6	0–6	≤2
	(ii) Perseverative errors	1 (1)	—	≥6.41
	Cognitive estimates			
	(i) Errors	19*	0–42	≥18
	(ii) Bizarre errors	4*	0–21	≥4

Praxis	Figure copying	9 (9.1)	0–12	≤7.18
	Rey-Osterrieth complex figure	31 (30.25)	0–36	≤28.87
	Clock test	9	0–10	≤7

Visual processing	Minimal feature view task	25	0–25	

**Table 2:** DA performance on selected motor tasks (the star indicates scores below cutoffs).

Motor tasks	Range	Cutoffs	Right hand raw score (corrected)	Left hand raw score (corrected)	R & L hand
Writing to dictation^1^					
(i) Words	0–10	≤6.4	10	7 (6.3)*	—
(ii) Nonwords	0–5	≤1.4	5	4 (3.3)	—
(iii) Sentences	0–2	<0.6	2	0*	—
Writing (copying)					
(i) Words	0–5	—	5	5	—
(ii) Digits	0–10	—	10	9	—
Action imitation^2^					
(i) MF actions	0–36	≤32	32	31*	—
(ii) ML actions	0–36	≤31	30*	30*	—
Tool use					
(i) Pantomimes (verbal)^3^	0–16	≤12	16	16	—
(ii) Single object use (a)^4^	0–14	<14	14	14	—
(iii) Single object use (b)	0–15	—	15	15	—
(iv) Multiple object use^5^	0–5	<5	—	—	5
Construction ability					
(i) Figure copying^6^	0–12	≤7.18	10 (10.1)	9/12 (9.1)	
(ii) Rey-Osterrieth complex fig.^7^	0–36	≤28.87	31		
(iii) WAIS-R block design^8^	0–36	—	—	—	22 (*z* = +0.0)
(iv) Clock drawing test^9^	0–10	≤7	9		
Tactile gnosis	0–20	—	19	16	—

Note: the different motor components were explored by mean of the following standardized tests: the writing subtests of the Esame Neuropsicologico per l'Afasia(Capasso, Miceli 2001)^1^; test for ideomotor apraxia (Tessari et al., 2011 [10])^2^; the pantomime subtest of the Limb Apraxia Battery (Bartolo et al., 2008)^3^; real object use in addition to ideational apraxia testing (DeRenzi et al., 1968)^4^; multiple object use (De Renzi and Lucchelli, 1988)^5^; the constructional apraxia subtest of the mental deterioration battery (Carlesimo et al., 1995)^6^; the Italian version of the Rey Figure copying test (Caffarra e coll., 2002)^7^; the Italian version of the block design subtest of the Wechsler adult intelligence scale (WAIS-R)^8^; clock drawing subtest of the Esame Neuropsicologico Breve (Mondini et al., 2008)^9^.
